# Polymyalgia rheumatica

**DOI:** 10.1007/s00393-026-01859-5

**Published:** 2026-08-31

**Authors:** Myriam Reisch, Zhivana Boyadzhieva, Christian Dejaco, Frank Buttgereit

**Affiliations:** 1 Abteilung für Rheumatologie und Immunologie, Innere Medizin, Medizinische Universität Graz, Graz, Österreich; 2 Medizinische Klinik m. S. Rheumatologie und Klinische Immunologie, Charité – Universitätsmedizin Berlin, Berlin, Deutschland; 3 Abteilung für Rheumatologie, Krankenhaus Bruneck (ASAA-SABES), Bruneck, Italien; 4 Abteilung für Rheumatologie, Lehrkrankenhaus der Paracelsus Medizinischen Privatuniversität, Bruneck, Italien; 5 Deutsches Rheumaforschungszentrum, ein Institut der Leibniz-Gemeinschaft, Berlin, Deutschland

**Keywords:** Rheumatische Erkrankungen, Differenzialdiagnosen, Risikobewertung, Interleukin-6-Rezeptor, Glukokortikoide, Rheumatic diseases, Differential diagnoses, Risk assessment, Interleukin‑6 receptor, Glucocorticoids

## Abstract

Die Polymyalgia rheumatica (PMR) ist eine häufige entzündlich-rheumatische Erkrankung im höheren Lebensalter, die durch bilaterale Schmerzen im Schulter- und Beckengürtel, Morgensteifigkeit sowie systemische Entzündungszeichen charakterisiert ist. Trotz ihrer Häufigkeit bleibt die PMR angesichts diagnostischer Unsicherheiten mit einem breiten Spektrum an Differenzialdiagnosen eine klinische Herausforderung. Zusätzlich erschweren die Überlappung mit der Riesenzellarteriitis sowie die aufgrund wiederholter Rezidive häufig erforderliche langfristige Glukokortikoidtherapie, die mit einem erhöhten Risiko für therapieassoziierte Nebenwirkungen einhergeht, das Management der Erkrankung. Entscheidend für die optimale Versorgung der Betroffenen ist eine frühzeitige Diagnosesicherung, eine strukturierte Patient:innenstratifizierung sowie das Einbeziehen individueller Risikofaktoren in die Therapieentscheidung.

## Abstract (en)

Polymyalgia rheumatica (PMR) is a common inflammatory rheumatic disease of older adults, characterized by bilateral pain in the shoulder and pelvic girdles, morning stiffness, and systemic signs of inflammation. Despite its high prevalence, PMR remains a clinical challenge due to diagnostic uncertainty and a broad spectrum of differential diagnoses. Furthermore, disease management is complicated by the overlap with giant cell arteritis and the frequently required long-term glucocorticoid therapy due to recurrent relapses, which in turn is associated with an increased risk of treatment-related adverse effects. Early confirmation of the diagnosis, structured patient stratification, and the integration of individual risk factors into therapeutic decision-making are essential for the optimal management of patients with PMR.

## Lernziele

Nach Lektüre dieses Beitrags sind Sie in der Lage ...

- die typischen klinischen Symptome der Polymyalgia rheumatica (PMR) zu benennen und diese korrekt in das Krankheitsbild einzuordnen.
- relevante diagnostische Befunde aus Labor, Klinik und Bildgebung zu identifizieren und die PMR von wichtigen Differenzialdiagnosen abzugrenzen.
- die klinische und pathogenetische Verbindung zwischen PMR und Riesenzellarteriitis zu erkennen daraus Konsequenzen für Diagnostik und Management abzugrenzen.
- die zentralen Therapieprinzipien der PMR in der klinischen Praxis umzusetzen.
- prognostische Faktoren und Aktivitätsparameter hinsichtlich ihres Nutzens für die Risikostratifizierung und Therapieanpassung zu beurteilen.

## Einleitung

Die Polymyalgia rheumatica (PMR) gehört zu den häufigsten entzündlich-rheumatischen Erkrankungen. Sie tritt i. d. R. erst ab dem 50. Lebensjahr auf, wobei das mittlere Alter bei Diagnosestellung zwischen 70 und 80 Jahren liegt. Die jährliche Inzidenz beträgt bei Personen über 50 Jahren bis zu 113 Fälle pro 100.000. Frauen sind dabei etwa zwei- bis dreimal häufiger betroffen als Männer [1].

Das Fehlen von diagnostischen Tests, das breite Spektrum an **Differenzialdiagnosen**

Differenzialdiagnosen,

häufige **Rezidive**

Rezidive

und die oft sehr lange Behandlung mit Glukokortikoiden, die mit entsprechenden **Nebenwirkungen**

Nebenwirkungen

verbunden ist, machen die PMR sowohl zur diagnostischen als auch zur therapeutischen Herausforderung.

Im April 2025 wurde eine aktualisierte deutschsprachige S2e-Leitlinie zur Behandlung der PMR publiziert. Im Vergleich zur vorherigen S3-Leitlinie aus dem Jahr 2018 stellt sie eine evidenzbasierte Aktualisierung auf Basis einer moderaten Evidenz und eines Expert:innenkonsenses dar, während S3-Leitlinien durch eine höhere Evidenzstärke sowie einen umfassenderen methodischen Konsens charakterisiert sind. Die Empfehlungen der S2e-Leitlinie bilden die Grundlage für die nachfolgend dargestellten Behandlungsoptionen [2].

## Immunpathophysiologie

Die PMR gilt als immunvermittelte entzündliche Erkrankung, deren Pathogenese auf einem komplexen Zusammenspiel altersassoziierter, genetischer und umweltbedingter Faktoren sowie einer Dysregulation der angeborenen und adaptiven Immunität beruht. Im Rahmen der **Immunseneszenz**

Immunseneszenz

kommt es zu einer verminderten Produktion naiver T‑Zellen, insbesondere **regulatorischer T‑Zellen**

regulatorischer T‑Zellen,

mit konsekutiver Störung der **Immunhomöostase**

Immunhomöostase.

Gleichzeitig fördern altersbedingte Defekte der Autophagie die Akkumulation **proinflammatorischer Zytokine**

proinflammatorischer Zytokine

und reaktiver Sauerstoffspezies im Sinne des **„inflammaging“**

„inflammaging“.

Immunologisch stellt **Interleukin‑6**

Interleukin‑6

(IL-6) den bislang am besten untersuchten und vermutlich zentralen pathogenetischen Mediator dar, insbesondere über die Aktivierung des JAK/STAT-Signalwegs. Erhöhte Spiegel weiterer proinflammatorischer Zytokine wie IL-1β, Tumornekrosefaktor‑α und IL-17 wurden ebenfalls beschrieben, wobei die Evidenz hierfür weniger konsistent ist. Zudem zeigt sich eine Polarisation von T‑Zellen in Richtung TH1- und insbesondere TH17-Phänotyp mit verstärkter IL-17-Produktion, wodurch Makrophagen, Endothel- und glatte Muskelzellen aktiviert und chronische Entzündungsprozesse perpetuiert werden. Interferon‑γ scheint hingegen stärker mit der Riesenzellarteriitis (RZA) assoziiert zu sein, die oft mit der PMR überlappt. Parallel zu den oben genannten Anomalien finden sich bei der PMR Veränderungen der angeborenen Immunität mit erhöhten Zellzahlen von neutrophilen Granulozyten und Monozyten. Auch die Rolle der B‑Zellen erscheint komplex und betrifft weniger die absolute Zellzahl als vielmehr funktionelle Veränderungen von regulatorischen und Effektor-B-Zell-Subpopulationen sowie deren Beitrag zur Produktion proinflammatorischer Zytokine, insbesondere von IL‑6. Auf Gewebeebene induzieren proinflammatorische Zytokine über die Aktivierung fibroblastenähnlicher Synoviozyten eine Entzündung in der Synovialis von Gelenken und Bursen mit einer sich selbst verstärkenden Produktion von IL‑6 und konsekutiver Aufrechterhaltung der inflammatorischen Kaskade [3].

## Diagnostik

Die Diagnosestellung der PMR basiert primär auf der klinischen Symptomatik in Kombination mit erhöhten Entzündungswerten sowie ggf. einer Bildgebung und erfordert eine sorgfältige **differenzialdiagnostische Evaluation**

differenzialdiagnostische Evaluation,

da zahlreiche entzündliche und nichtentzündliche Erkrankungen ein vergleichbares klinisches Erscheinungsbild verursachen können. Differenzialdiagnostisch abzugrenzen sind insbesondere andere entzündlich-rheumatische Erkrankungen, z. B. „elderly-onset rheumatoid arthritis“ (EORA), Calciumpyrophosphat-Dihydrat(CPPD)-Arthropathie, Spondyloarthritis, entzündliche Myopathien, sowie degenerative Ursachen, z. B. aktivierte bilaterale Omarthrosen, außerdem paraneoplastische, infektiöse, endokrinologische und medikamentenassoziierte Krankheitsbilder (Tab. 1). Daten aus der Primärversorgung weisen darauf hin, dass die initiale Diagnose PMR im Verlauf häufig korrigiert werden muss [4]. Dies verdeutlicht die Wichtigkeit einer frühzeitigen fachärztlich-rheumatologischen Evaluation zur bestmöglichen Sicherung der Diagnose.

**Tab. 1:** Differenzialdiagnosen der Polymyalgia rheumatica (PMR)

Differenzialdiagnose	Unterschiede zur klassischen PMR-Symptomatik
EORA	Synovitis kleiner Gelenke (MCP/PIP), erosive Veränderungen, *RF-/ACPA-Positivität*, ausgeprägte periphere Arthritis
CPPD-Arthropathie	Akute Attacken, intermittierende Verläufe, Knie‑/Handgelenkbeteiligung, starke Nackenschmerzen, *Chondrokalzinose in Bildgebung*
Spondyloarthritis	Entzündlicher Rückenschmerz, *Enthesitiden, Daktylitis, Psoriasis, Uveitis, chronisch-entzündliche Darmerkrankung*, HLA-B27-Assoziation
Entzündliche Myopathien	Proximale *Muskelschwäche* statt Schmerzdominanz, CK-Erhöhung, pathologisches EMG/MRT, Hautmanifestationen
Degenerative Schultergürtelerkrankungen	*Belastungsabhängige Schmerzen*, fehlende systemische Entzündungszeichen, normale Entzündungsparameter, strukturelle Veränderungen in Sonographie/MRT/Röntgen
Osteoarthritis	Mechanischer Schmerzcharakter, kurze Morgensteifigkeit (< 30 min), typische *radiologische Arthrosezeichen*
Paraneoplastische Syndrome	B‑Symptomatik, Gewichtsverlust, atypische oder refraktäre Beschwerden, oft *inkomplettes Ansprechen auf die initiale Glukokortikoidtherapie*, Anämie oder andere auffällige Laborbefunde
Infektionen	*Fieber, Infektfokus*, deutlich erhöhte Entzündungsparameter, fehlendes Ansprechen auf Glukokortikoide
Endokrinologische Erkrankungen (z. B. Hypothyreose, Hyperparathyreoidismus)	Myalgien ohne klare Synovitis/Bursitis, *metabolische Auffälligkeiten, CK-/TSH-/Kalziumveränderungen*
Medikamentenassoziierte Syndrome (z. B. statinassoziierte Myopathie)	*Zeitlicher Zusammenhang mit Therapiebeginn*, Myopathiezeichen, CK-Erhöhung, atypische Verteilung der Beschwerden
Fibromyalgie	*Generalisierte Schmerzsymptomatik*, Fatigue, Schlafstörungen, vegetative Begleitsymptome, fehlende objektive Entzündungszeichen
Neurologische Erkrankungen (z. B. Parkinson-Syndrom)	*Rigor, Bradykinese, Gangstörung*, fehlende inflammatorische Konstellation

*PMR* Polymyalgia rheumatica, *EORA* „elderly-onset rheumatoid arthritis“, *MCP* Metakarpophalangealgelenk, *PIP* proximales Interphalangealgelenk, *RF* Rheumafaktor, *ACPA* Antikörper gegen citrullinierte Peptide, *CPPD* Calciumpyrophosphat-Dihydrat, *HLA-B27* humanes Leukozytenantigen B27, *MRT* Magnetresonanztomographie, *CK* Kreatinkinase, *EMG* Elektromyographie, *TSH* Thyreoidea stimulierendes Hormon

### Klinik

Die PMR manifestiert sich klinisch überwiegend durch bilaterale Schmerzen im Bereich des Schultergürtels und des Nackens, der proximalen Oberarme sowie der Hüfte. Charakteristisch ist eine **symmetrische Schmerzverteilung**

symmetrische Schmerzverteilung,

während mechanisch-degenerativ bedingte Schmerzen meist unilateral dominiert sind [5].

Bei der PMR wird typischerweise auch eine **ausgeprägte Morgensteifigkeit**

ausgeprägte Morgensteifigkeit

mit einer Dauer von mindestens 30–45 min beobachtet. Der Krankheitsbeginn ist i. d. R. akut bis subakut und kann mit **Allgemeinsymptomen**

Allgemeinsymptomen

wie Abgeschlagenheit, subfebrilen Temperaturen und ungewolltem Gewichtsverlust einhergehen [6]. In einigen Fällen treten **periphere Arthritiden**

periphere Arthritiden

auf, was die Differenzierung gegenüber der seronegativen EORA erschweren kann [7]. Zudem kann bei einem Teil der Patient:innen mit initial diagnostizierter PMR sich im Verlauf eine EORA manifestieren bzw. die initiale Diagnose einer PMR reklassifiziert werden. In longitudinalen Kohortenstudien wurde eine entsprechende Umklassifizierung bei etwa 10 % der Patient:innen beschrieben [8].

### Labordiagnostik

Derzeit stehen für die PMR keine spezifischen laborchemischen Biomarker in der klinischen Routine zur Verfügung. Typischerweise findet sich eine Erhöhung der **Blutsenkungsgeschwindigkeit**

Blutsenkungsgeschwindigkeit

(BSG) und/oder des **C‑reaktiven Proteins**

C‑reaktiven Proteins

(CRP), wobei in seltenen Fällen (ca. 1–3 %) auch eine aktive PMR mit normalen Entzündungsparametern beschrieben wurde [9].

#### Merke

Normale Entzündungswerte machen eine PMR sehr unwahrscheinlich, schließen sie aber nicht vollständig aus.

Zusätzlich können als Zeichen der systemischen Entzündung eine milde normochrome, normozytäre Anämie, Thrombozytose, Hypoalbuminämie sowie erhöhte Fibrinogenspiegel auftreten. Bei bis zu einem Drittel der Patient:innen findet sich zudem eine leichte Erhöhung der **alkalischen Phosphatase**

alkalischen Phosphatase

[10].

Die aktuell gültige S2e-Leitlinie zur Behandlung der PMR empfiehlt zur Erstdiagnostik neben BSG und CRP auch die Erhebung eines kompletten Blutbildes sowie von Elektrolyten, Blutzucker, Leber- und Nierenparametern, da diese Werte für die **Verlaufskontrolle**

Verlaufskontrolle

und das Monitoring unter **immunsuppressiver Therapie**

immunsuppressiver Therapie

von Bedeutung sind. Zur differenzialdiagnostischen Abklärung sollten zusätzlich der Rheumafaktor, Anti-CCP-Antikörper, die Serumelektrophorese mit Immunfixation, das Thyreoidea stimulierende Hormon (TSH) sowie die Kreatinkinase (CK) bestimmt werden. Bei entsprechender klinischer Symptomatik ist außerdem eine Testung auf antinukleäre Antikörper (ANAs) und antineutrophile zytoplasmatische Antikörper (ANCAs) sinnvoll, um andere entzündlich-rheumatische Erkrankungen auszuschließen.

Für die Erfassung der Krankheitsaktivität im Verlauf dienen primär CRP und BSG als **serologische Marker**

serologische Marker.

Diese sind jedoch unter einer Therapie mit **IL-6-Rezeptor-Inhibitoren**

IL-6-Rezeptor-Inhibitoren

nicht aussagekräftig, weil die IL-6-vermittelte hepatische **Akute-Phase-Reaktion**

Akute-Phase-Reaktion

gehemmt wird und dadurch insbesondere das CRP trotz persistierender Krankheitsaktivität (aber auch bei Infektionen) nicht erhöht ist.

Derzeit werden verschiedene andere Biomarker wie **Serumamyloid A**

Serumamyloid A

(SAA), Calprotectin, Osteopontin, aber auch der Neutrophilen-Lymphozyten- oder der Thrombozyten-Lymphozyten-Quotient zur verbesserten Diagnostik und Aktivitätsbeurteilung der PMR untersucht [11]. Das SAA könnte eine sensitivere Detektion der entzündlichen Aktivität bei PMR als die BSG und das CRP ermöglichen [12]. Zur Identifikation einer subklinischen Vaskulitis bei PMR-Patient:innen sind die vaskulären Remodellierungsmarker Angiopoietin‑2 und MMP‑3 vielversprechend [13].

### Bildgebung

**Abb. 1:**
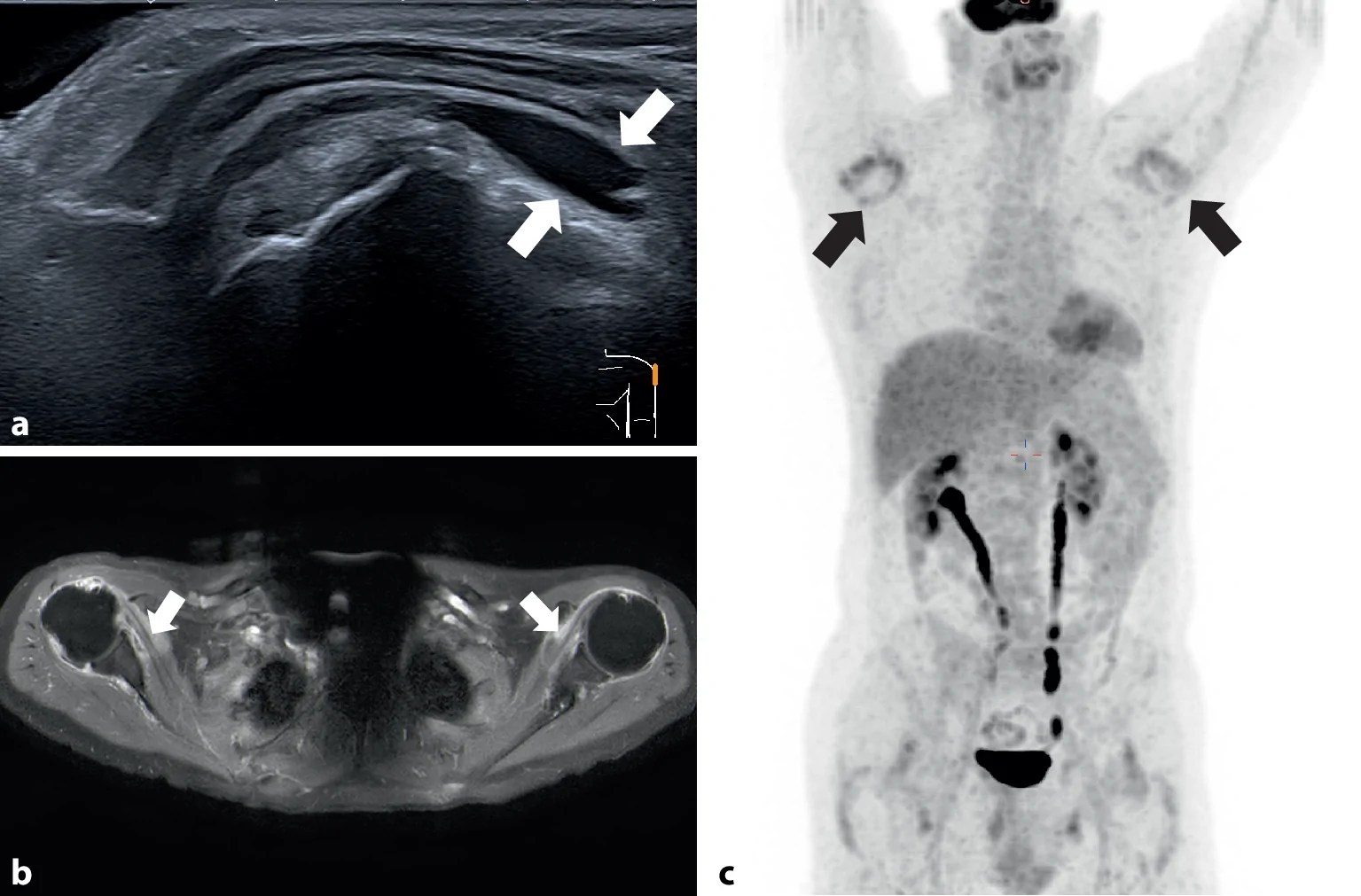
Beispiele für die Ergebnisse bildgebender Verfahren bei der Polymyalgia rheumatica. **a** Ultraschallbild (nur Grauskala) eines Längsschnitts der Schulter einer Patientin. Die *gefüllten Pfeile* weisen auf eine Bursitis subdeltoidea hin. **b** Magnetresonanztomogramm beider Schultern, die *offenen Pfeile* zeigen eine Kontrastmittelaufnahme entlang des Sehnen-Muskel-Übergangs des M. subscapularis. **c** Bild einer Fluor-18-Desoxyglukose-Positronenemissionstomographie, bei der eine Anreicherung des Tracers an den Schultergelenken zu sehen ist. (Mit freundlicher Genehmigung, © Dr. Tommaso Buzzegoli und Dr. Mohsen Farsad, alle Rechte vorbehalten)

Neben der klinischen und laborchemischen Diagnostik kommt der Bildgebung in ausgewählten Fällen, wie bei typischer PMR-Symptomatik trotz normwertiger Entzündungsparameter, eine wichtige diagnostische Bedeutung zu (Abb. 1; [14]).

#### Ultraschall

Ein typisches sonographisches Merkmal ist die (häufig beidseitige) **subakromial-subdeltoidale Bursitis**

subakromial-subdeltoidale Bursitis,

deren diagnostische Aussagekraft jedoch nur moderat ist (Sensitivität ca. 66 %, Spezifität ca. 89 %). Weitere Befunde wie synoviale Verdickungen oder Flüssigkeitsansammlungen um die lange Bizepssehne, beidseitige Bursitis trochanterica sowie Ergüsse oder Synovitiden im Glenohumeralgelenk können die Diagnose stützen, sind jedoch nicht spezifisch für die PMR [15].

Die kombinierte Untersuchung von Schulter- und Hüftregion kann die diagnostische Genauigkeit gegenüber der Untersuchung einer Einzelregion deutlich erhöhen [16]. Die Differenzierung der PMR von anderen entzündlichen Erkrankungen wie der EORA mittels Ultraschall ist schwierig, da letztere ähnliche sonographische Veränderungen verursacht (Spezifität ca. 70 %). Mechanisch bedingte Veränderungen, wie beispielsweise Läsionen der Rotatorenmanschette lassen sich hingegen mit einer höheren Genauigkeit (Spezifität ca. 89 %) abgrenzen [17].

#### Magnetresonanztomographie (MRT)

Im Vergleich zum Ultraschall ist die MRT teurer und technisch aufwendiger, weist aber auch eine höhere Sensitivität und Spezifität für den Nachweis PMR-typischer entzündlicher Veränderungen auf, v. a. im periartikulären Weichteilgewebe. Für die PMR charakteristische MRT-Befunde sind entzündliche Veränderungen im **peritendinösen Gewebe**

peritendinösen Gewebe

sowie an den **Muskel-Sehnen-Übergängen**

Muskel-Sehnen-Übergängen

des Schulter- und Beckengürtels. Werden beispielsweise im Bereich des Beckengürtels beidseits mindestens drei entzündlich veränderte Strukturen nachgewiesen, kann eine hohe diagnostische Sicherheit mit Sensitivitäten bis zu 100 % und Spezifitäten über 97 % erreicht werden [18, 19].

Im Gegensatz zur PET/CT (s. unten) erlaubt die MRT jedoch keine systemische Beurteilung des gesamten Körpers und ist zur Detektion einer vaskulären Mitbeteiligung im Rahmen der RZA nur eingeschränkt geeignet. Daher kann sie primär zur differenzialdiagnostischen Abklärung von strukturellen Schulter- oder Hüftpathologien dienen.

#### Fluor-18-FDG-PET/CT

Die Fluor-18-Fluordesoxyglucose-Positronenemissionstomographie kombiniert mit Computertomographie (^18^F‑FDG-PET/CT) ermöglicht eine umfassende Darstellung entzündlicher Prozesse im gesamten Körper. Charakteristisch für die PMR ist eine verstärkte **periartikuläre Anreicherung**

periartikuläre Anreicherung

von ^18^F‑FDG an Schultern und Hüften, gelegentlich auch an weiteren Strukturen wie den Tuberositates ischiadicae (Peritendinitis der dorsalen Oberschenkelmuskulatur) oder den interspinalen Bursen. Die PET/CT weist eine hohe Sensitivität (92,6 %) und Spezifität (90 %) für die Diagnose der PMR auf [20]. Aufgrund der **begrenzten Auflösung**

begrenzten Auflösung

sowie der **Strahlenexposition**

Strahlenexposition

ist sie jedoch keine Bildgebung der ersten Wahl. Ihr Einsatz wird insbesondere bei **atypischen Verläufen**

atypischen Verläufen

empfohlen, um Differenzialdiagnosen oder zugrunde liegende andere Erkrankungen wie die RZA oder Malignome auszuschließen [21]. Da eine PET/CT-Diagnostik im klinischen Alltag häufig nicht unmittelbar verfügbar ist und aufgrund der meist ausgeprägten Symptomatik i. d. R. nicht mit dem Beginn der Glukokortikoidtherapie zugewartet werden kann, ist die diagnostische Sensitivität häufig bereits eingeschränkt [22].

## Klassifikationskriterien

Im Jahr 2012 veröffentlichte die European Alliance of Associations for Rheumatology (EULAR) gemeinsam mit dem American College of Rheumatology (ACR) Klassifikationskriterien für die PMR. Diese sollten primär die standardisierte Aufnahme von Patient:innen in klinische Studien und Kohorten erleichtern. Nach Erfüllung der drei Eingangskriterien – Alter über 50 Jahre, neu aufgetretene bilaterale Schulterschmerzen sowie Erhöhung der Laborparameter CRP und/oder BSG – werden weitere klinische und laborchemische Parameter mittels eines Punktesystems bewertet (Tab. 2). Der sonographische Nachweis von PMR-spezifischen Läsionen kann zusätzliche Punkte ergeben. Eine Gesamtpunktzahl von mindestens 4 (ohne Ultraschall) bzw. 5 (mit Ultraschall) ermöglicht die Klassifikation als PMR. Die Einbeziehung der Sonographie verändert die Sensitivität (von 68 % auf 66 %) und Spezifität (von 78 % auf 81 %) der Klassifikationskriterien jedoch nur minimal [23].

**Tab. 2:** Klassifikationskriterien des American College of Rheumatology (ACR) und der European League Against Rheumatism (EULAR) zur Polymyalgia rheumatica. (Adaptiert nach den ACR/EULAR-Klassifikationskriterien zur Polymyalgia rheumatica 2012 [23])

*Verpflichtende Eingangskriterien*	Alter über 50 Jahre
	Neu aufgetretene bilaterale Schulterschmerzen
	Erhöhung der Laborparameter CRP und/oder BSG
*Kriterien (Punkteanzahl)*	Morgensteifigkeit > 45 min (2)
	Hüftschmerzen oder eingeschränkte Beweglichkeit der Hüfte (1)
	Keine periphere Gelenkbeteiligung (1)
	Fehlen von RF und/oder ACPA (2)
*Sonographie (Punkteanzahl)*	Nachweis einer subdeltoiden/subakromialen Bursitis und/oder synovialen Proliferation/Ergussbildung um die lange Bizepssehne und/oder Synovitis des Glenohumeralgelenks in einer Schulter und Synovitis und/oder Bursitis trochanterica in einer Hüfte (1)
	Nachweis einer subdeltoiden/subakromialen Bursitis und/oder synovialen Proliferation/Ergussbildung um die lange Bizepssehne und/oder Synovitis des Glenohumeralgelenks in beiden Schultern (1)
*Auswertung*	Ohne Sonographie: Gesamtscore ≥ 4 → Klassifikation als PMR möglich
	Mit Sonographie: Gesamtscore ≥ 5 → Klassifikation als PMR möglich

*PMR* Polymyalgia rheumatica, *CRP* C-reaktives Protein, *BSG* Blutsenkungsgeschwindigkeit, *RF* Rheumafaktor, *ACPA* Antikörper gegen citrullinierte Proteine/Peptide

Wichtig zu erwähnen ist, dass diese Klassifikationskriterien primär für **Forschungszwecke**

Forschungszwecke

entwickelt wurden und keine Diagnosekriterien darstellen. Das heißt, dass bei Patient:innen, die diese Kriterien nicht erfüllen, dennoch eine PMR diagnostiziert werden kann. Im klinischen Alltag bleibt deshalb die umfassende Beurteilung aus Anamnese, klinischer Untersuchung, Labor- und eventueller Bildgebung der Goldstandard für die Diagnostik.

## Prognosefaktoren

Die Datenlage zu prognostischen Faktoren bei einer neu diagnostizierten PMR ist heterogen und teilweise inkonsistent. Ob das **Lebensalter**

Lebensalter

einen möglichen Risikofaktor darstellt, ist unklar. So zeigen einzelne Studien keinen signifikanten Einfluss des Alters auf das Rezidivrisiko, während andere ein erhöhtes Rückfallrisiko bei Patient:innen über 60 Jahre beschreiben. Jüngere Betroffene entwickeln häufiger einen **glukokortikoidabhängigen Verlauf**

glukokortikoidabhängigen Verlauf,

bei dem es im Rahmen der Dosisreduktion zu Krankheitsrezidiven kommt. **Geschlechtsspezifische Effekte**

Geschlechtsspezifische Effekte

sind insgesamt nicht konsistent nachweisbar, wenngleich einzelne Arbeiten über eine niedrigere Remissionsrate bei Frauen berichten. Erhöhte CRP-Werte bei Diagnosestellung sowie eine **verzögerte CRP-Normalisierung**

verzögerte CRP-Normalisierung

sind mit einem erhöhten Rezidivrisiko assoziiert, während die BSG als weniger verlässlicher Prädiktor gilt. Ergänzend werden die Erythrozytenverteilungsbreite und der Neutrophilen-Lymphozyten-Quotient als potenzielle Marker für frühe Rezidive bzw. Glukokortikoidresistenz diskutiert. Klinisch zeigt das Vorliegen einer peripheren Arthritis keinen konsistenten Einfluss auf Rezidivraten, während eine längere Symptomdauer vor Diagnosestellung mit einem geringeren Rezidivrisiko assoziiert ist [24].

## Bestimmung der Krankheitsaktivität

Das primäre Behandlungsziel der PMR ist die **klinische Remission**

klinische Remission,

die als das vollständige Verschwinden der Symptomatik sowie die Normalisierung der Entzündungswerte definiert wird [25].

Trotz der Behandlung mit Glukokortikoiden erleiden etwa 30–40 % der Patient:innen mit PMR im 1. Jahr ein Rezidiv. Auch die Rückfallhäufigkeit über 5 Jahre beträgt bis zu 60 %. Ein Rezidiv äußert sich meist durch die erneute Zunahme der charakteristischen Schmerzen und Morgensteifigkeit sowie durch einen Anstieg der Entzündungsmarker [26].

Für die Bewertung der Krankheitsaktivität in klinischen Studien und der Praxis wird häufig der **PMR-Aktivitätsscore**

PMR-Aktivitätsscore

(PMR-AS) eingesetzt [27]. Dieser setzt sich zusammen aus den vier klinischen Parametern Schmerzintensität, Einschätzung der Krankheitsaktivität durch die Untersucher:innen, Dauer der Morgensteifigkeit und Beweglichkeit des Schultergürtels („elevation of upper limb“, EUL) sowie dem CRP (alternativ der BSG; Tab. 3) . Der PMR-AS ermöglicht eine **standardisierte Beurteilung**

standardisierte Beurteilung

des Krankheitsverlaufs, erleichtert die frühzeitige Erkennung von Rückfällen und unterstützt die individuelle Anpassung der Therapie. Er ist mittlerweile in Studien validiert, wird jedoch im klinischen Alltag bislang nur selten routinemäßig verwendet. Zudem besteht weiterhin Uneinigkeit hinsichtlich der **Cut-offs**

Cut-offs

für die verschiedenen Krankheitsaktivitätsstadien. Während in klinischen Studien häufig ein Wert < 10 Punkten als Surrogat für Remission verwendet wird, liegen die ursprünglich vorgeschlagenen Grenzwerte einer Remission bei < 1,5 bzw. einer niedrigen Krankheitsaktivität bei < 7. Limitationen bestehen zudem in der eingeschränkten Spezifität einzelner Parameter (z. B. der Beweglichkeit des Schultergürtels), der fehlenden Berücksichtigung der Intensität der Morgensteifigkeit sowie möglichen Fehlinterpretationen durch Komorbiditäten. Unter einer durch IL-6-Rezeptor-Inhibition bedingten Suppression von CRP und BSG ist der Score zudem nur eingeschränkt interpretierbar. Ein CRP-freier klinischer PMR-AS wurde zwar bereits entwickelt, ist jedoch noch nicht ausreichend validiert [28].

**Tab. 3:** Bestimmung der Krankheitsaktivität anhand des Polymyalgia-rheumatica-Aktivitätsscores (PMR-AS). (Adaptiert nach dem PMR-AS von Leeb u. Bird [27])

PMR-AS=CRP (mg/dl) + VAS „pain“ (0–10) + VAS „phass“ (0–10) + (MST [min] × 0,1) + EUL (3–0)	
*Beurteilung:*	Remission: 0 bis 1,5 Punkte
	Niedrige Krankheitsaktivität: < 7 Punkte
	Mittlere Krankheitsaktivität: 7 bis 17 Punkte
	Hohe Krankheitsaktivität: > 17 Punkte
	Rezidiv: ΔPMR-AS ≥ 6,6 Punkte, PMR-AS ≥ 9,35 Punkte

*CRP* C-reaktives Protein, *VAS* visuelle Analogskala, *pain* Schmerz (0 = kein Schmerz bis 10 = nichtauszuhaltender Schmerz), *„phass“* Krankheitsaktivität (Beurteilung durch Untersucher:innen), *MST* Morgensteifigkeit, *min* Minuten, *EUL* „elevation of upper limb“ (Fähigkeit, die Arme zu bewegen: 0 = über Schulterhöhe, 1 = bis Schulterhöhe, 2 = unterhalb Schulterhöhe, 3 = keine Elevationsbewegung möglich)

## PMR und Riesenzellarteriitis als Spektrumerkrankung

Die RZA ist eine **Großgefäßvaskulitis**

Großgefäßvaskulitis,

die bevorzugt die extrakraniellen Äste der A. carotis sowie die Aorta und deren große Abgänge betrifft und die häufigste primäre Vaskulitis der Erwachsenen darstellt. Typische klinische Manifestationen des kranialen Gefäßbefalls sind u. a. Kopfschmerzen, Visusstörungen und Kauschmerzen. Bei bis zu 65 % der Patient:innen mit RZA bestehen gleichzeitig Symptome einer PMR. Umgekehrt entwickelt sich bei etwa 10–20 % der Patient:innen mit initialer Diagnose einer isolierten PMR im Verlauf eine RZA. Diese bidirektionale Überlappung unterstreicht die enge klinische und **pathogenetische Verknüpfung**

pathogenetische Verknüpfung

beider Entitäten, die als PMR-RZA-Spektrum verstanden wird [29, 30].

Bildgebend kann mittels Ultraschall bei bis zu 22,8 % der Patient:innen mit isolierter PMR eine subklinische RZA nachgewiesen werden. Diese Patient:innen benötigten höhere kumulative Glukokortikoiddosen und entwickelten häufiger Rezidive. Dieser Zusammenhang konnte in PET/CT-Studien jedoch nicht konsistent bestätigt werden, weshalb derzeit kein systematisches Screening von PMR-Patient:innen hinsichtlich entzündlicher Beteiligung der großen Gefäße empfohlen wird [31].

Daher sollte insbesondere bei persistierender systemischer Inflammation oder bildgebenden Hinweisen die Diagnose einer RZA frühzeitig erwogen werden, um eine verzögerte Diagnosestellung zu verhindern [32].

### Merke

Bei persistierend erhöhten Entzündungsparametern trotz Glukokortikoidtherapie sollte differenzialdiagnostisch an eine Riesenzellarteriitis gedacht werden.

## Immuncheckpoint-Inhibitor-assoziierte Polymyalgia rheumatica

Immuncheckpoint-Inhibitoren (ICI) haben die Therapie zahlreicher maligner Erkrankungen revolutioniert und finden zunehmend klinische Anwendung. Als **monoklonale Antikörper**

monoklonale Antikörper

gegen CTLA‑4, PD‑1 oder PD-L1 verstärken sie durch die Blockade inhibitorischer Immuncheckpoints die antitumorale T‑Zell-Antwort. Im Rahmen der ICI-Therapie sind zahlreiche immunvermittelte Nebenwirkungen („immune-related adverse events“) beschrieben worden, wie v. a. Kolitis, Thyreoiditis und Hepatitis. Außerdem wurde auch von PMR-ähnlichen Syndromen mit einer Inzidenz von 2–21 pro 1000 Patient:innen unter ICI-Therapie berichtet. Ob es sich hierbei um eine echte therapieinduzierte Nebenwirkung oder um eine zufällige Koinzidenz handelt, wird kontrovers diskutiert. Die ICI-assoziierte PMR zeigt dabei einen milderen Verlauf als die klassische PMR, mit geringerer Morgensteifigkeit und niedrigerer BSG. Zudem erfüllten diese Patient:innen seltener die EULAR/ACR-Klassifikationskriterien (66,7 % vs. 97,3 %) und benötigten geringere Glukokortikoiddosen, die meist schneller reduziert werden konnten [33, 34].

## Management und Therapie der PMR

Die PMR verursacht i. d. R. keine bleibenden strukturellen Schäden und spricht häufig gut auf eine Glukokortikoidtherapie an, jedoch können die Diagnostik und **Langzeittherapie**

Langzeittherapie

herausfordernd sein.

Einer internationalen Umfrage zufolge werden lediglich ca. 25 % der Patient:innen mit PMR einer **rheumatologischen Fachabklärung**

rheumatologischen Fachabklärung

zugewiesen. Ein wesentliches Problem für die zeitnahe Zuweisung stellen dabei lange Wartezeiten auf fachärztliche Termine dar, sodass bei bis zu 50 % der Patient:innen bereits vor einer rheumatologischen Beurteilung eine Glukokortikoidtherapie eingeleitet wird, was die diagnostische Einordnung erheblich erschwert [35]. Beobachtungsstudien zeigen, dass etwa ein Drittel der Fälle im Verlauf mit einer neuen Diagnose klassifiziert wird [36].

Das vorrangige Ziel der Behandlung bei PMR besteht darin, eine Remission zu erreichen und dauerhaft mit einem Minimum an Medikamenten, insbesondere Glukokortikoiden, aufrechtzuerhalten. Die Therapieentscheidung sollte gemeinsam im **partizipativen Dialog**

partizipativen Dialog

zwischen behandelnden Ärzt:innen und Patient:innen getroffen werden. **Schulungen**

Schulungen

zu Erkrankung und Therapie werden ergänzend empfohlen. Zur Diagnosesicherung, Risikobewertung und Therapiefestlegung sollten Betroffene gemäß der S2e-Leitlinie zeitnah an eine rheumatologische Fachperson überwiesen werden. Die weitere Betreuung, insbesondere unter alleiniger Glukokortikoidtherapie, kann anschließend auch hausärztlich erfolgen. Bei Einsatz von krankheitsmodifizierenden Antirheumatika (DMARDs) wie der IL-6-Rezeptor-Blockade wird eine weiterführende Betreuung durch Fachärzt:innen empfohlen. Während der Anfangsphase der Behandlung sind Kontrolluntersuchungen in Intervallen von 7 bis 28 Tagen ratsam, um die Wirksamkeit der Therapie zu überprüfen und mögliche Nebenwirkungen frühzeitig zu erkennen. Sobald eine stabile Remission erreicht ist, können die Untersuchungsintervalle auf 3 bis 6 Monate ausgeweitet werden. Die Verlaufskontrollen beinhalten die Erfassung der Krankheitsaktivität anhand klinischer und laborchemischer Parameter, die Überwachung möglicher Nebenwirkungen sowie die Berücksichtigung von Begleiterkrankungen. Nach Abschluss der medikamentösen Behandlung können Verlaufskontrollen individuell angepasst werden. Eine definierte **Nachbeobachtungsdauer**

Nachbeobachtungsdauer

oder ein etabliertes Kriterium für eine dauerhafte Heilung der PMR existiert bislang nicht. Bei länger andauernder klinischer Remission ohne erneute Krankheitsaktivität kann jedoch von einer anhaltenden Remission ausgegangen werden. In diesem Fall kann Patient:innen nach entsprechender Aufklärung eine bedarfsweise Vorstellung angeboten werden [2].

Aufgrund der lang andauernden Glukokortikoidtherapie sollten eine **Osteoporoseprophylaxe**

Osteoporoseprophylaxe

inklusive individueller Risikobewertung und ggf. DXA-Screening sowie unter genereller immunsuppressiver Therapie ein adäquates **Impfmanagement**

Impfmanagement

und die Berücksichtigung von **Infektionsprophylaxen**

Infektionsprophylaxen

erfolgen.

### Medikamentöse Therapie

#### Glukokortikoide

Die initiale Therapie der PMR erfolgt i. d. R. mit Glukokortikoiden in einer morgendlichen Einmaldosis von 15–25 mg **Prednisonäquivalent**

Prednisonäquivalent

pro Tag. Initialdosen unter 7,5 mg/Tag werden aufgrund unzureichender Wirksamkeit nicht empfohlen, während Dosen über 30 mg/Tag keinen zusätzlichen therapeutischen Nutzen zeigen, jedoch mit einem erhöhten Risiko für unerwünschte Wirkungen assoziiert sind. Die **Dosisreduktion**

Dosisreduktion

sollte unter engmaschiger klinischer und laborchemischer Verlaufskontrolle schrittweise erfolgen. Innerhalb der ersten 4 bis 8 Wochen ist eine Reduktion auf etwa 10 mg/Tag anzustreben. Anschließend erfolgt eine weitere langsame Dosisreduktion, typischerweise um ca. 1 mg alle 4 Wochen, bis zum Therapieende. Im Falle eines Rezidivs sollte die Glukokortikoiddosis vorübergehend auf das Niveau vor dem Rückfall eskaliert und anschließend erneut schrittweise reduziert werden. Bei unkompliziertem Verlauf sollte die Gesamtdauer der Glukokortikoidmonotherapie 12 Monate nicht überschreiten. Real-World-Daten zeigen, dass die Glukokortikoidtherapie bei PMR häufig über mehrere Jahre fortgeführt wird. Nach 5 Jahren erhielten noch etwa 25 % der Patient:innen Glukokortikoide [26]. Die damit verbundene **prolongierte Exposition**

prolongierte Exposition

geht mit einer **relevanten Morbidität**

relevanten Morbidität

einher. So wurden innerhalb des 1. Jahres nach Beginn dieser Therapie bei 49 % der Patient eine Osteoporose, bei 30,2 % ein Diabetes mellitus und bei 14,9 % eine arterielle Hypertonie dokumentiert, wobei die Häufigkeit glukokortikoidassoziierter Komplikationen im weiteren Verlauf noch zunahm [37]. Der Einsatz einer begleitenden immunsuppressiven bzw. **steroidsparenden Therapie**

steroidsparenden Therapie

kann eine raschere Reduktion und somit eine Verkürzung dieser Glukokortikoidexposition ermöglichen (Tab. 4; [2]).

**Tab. 4:** Übersicht zu medikamentösen Therapieoptionen bei Polymyalgia rheumatica. (Therapieschema adaptiert nach der S2e-Leitlinie zur Behandlung der Polymyalgia rheumatica 2025 [2])

Initialtherapie mit Glukokortikoid	15–25 mg Prednisonäquivalent pro Tag
Reduktionsschema der Glukokortikoidtherapie	Innerhalb von 4 bis 8 Wochen Reduktion auf 10 mg/d Prednisonäquivalent, dann schrittweise Reduktion um 1 mg alle 4 Wochen bis zum Absetzen (Therapiedauer beträgt ca. 1 Jahr)
Therapie des Rezidivs	Erhöhung zumindest auf die Prärezidivdosis mit anschließender schrittweiser Dosisreduktion über 4 bis 8 Wochen wieder auf die ursprüngliche Dosis, bei der das Rezidiv auftrat
Therapieerweiterung bei hohem Risiko für glukokortikoidinduzierte Nebenwirkungen oder Rezidiven	IL-6-Rezeptor-Inhibitoren
	Alternative: Methotrexat, Secukinumab, Rituximab
Verkürzung des Glukokortikoidtherapieschemas unter DMARDs	Bei IL-6-Rezeptor-Inhibitoren und Rituximab: ca. 4 Monate
	Bei Methotrexat: ca. 6 bis 8 Monate

*DMARDs* „disease-modifying anti-rheumatic drugs“, *IL* Interleukin

#### Krankheitsmodifizierende Antirheumatika (DMARDs)

Bei Patient:innen mit einem erhöhten Risiko für glukokortikoidbedingte Nebenwirkungen oder Rezidiven kann von Beginn an als **Off-label-Therapie**

Off-label-Therapie

eine Kombinationstherapie mit IL-6-Rezeptor-Inhibitoren wie **Sarilumab**

Sarilumab

oder Tocilizumab in Erwägung gezogen werden. Bislang ist jedoch nur Sarilumab für die Behandlung von Patient:innen mit bereits rezidivierender oder refraktärer PMR zugelassen. Randomisierte kontrollierte Studien konnten eine effektive Kontrolle der Krankheitsaktivität, Reduktion der kumulativen Glukokortikoiddosis sowie Rezidivhäufigkeit durch die Blockade des IL-6-Rezeptors zeigen [38, 39].

Die aktualisierte S2e-Leitlinie bewertet den Stellenwert von **Methotrexat**

Methotrexat

gegenüber IL‑6 Rezeptor-Inhibitoren nun zurückhaltender. Methotrexat bleibt zwar eine mögliche Behandlungsalternative, jedoch war und ist die Evidenzbasis hierfür uneinheitlich. In speziellen Fällen, beispielsweise bei vorbestehender Divertikulitis oder ausgeprägter Nadelphobie, kann eine orale Methotrexatgabe als Alternative zur IL-6-Rezeptor-Blockade in Betracht gezogen werden. In Studien wurde Methotrexat in Dosierungen zwischen 7,5 mg und 25 mg verwendet, die Autor:innen setzen in der Praxis meist eine Dosis von 15 mg pro Woche ein. Höhere Dosierungen sind oft nicht möglich, da viele Patient:innen eine eingeschränkte Nierenfunktion aufweisen [24].

In der Phase-III-Studie REPLENISH zur Prüfung der Wirksamkeit von **Secukinumab**

Secukinumab

bei PMR erreichte ein signifikant höherer Anteil der Patient:innen unter Secukinumab eine anhaltende Remission in Woche 52 sowie eine niedrigere kumulative Glukokortikoiddosis als unter Placebo. Dies resultierte auch in einer geringeren Glukokortikoidtoxizität, die anhand des Glucocorticoid Toxicity Index gemessen wurde. Secukinumab könnte somit in Zukunft eine zugelassene Behandlungsoption für die PMR darstellen [40]. Zusätzlich wird **Rituximab**

Rituximab

als potenzielle Reserveoption diskutiert, gestützt auf eine randomisierte, placebokontrollierte Studie (BRIDGE-PMR) mit 47 Patient:innen, in der eine einmalige Rituximabgabe in Kombination mit Glukokortikoiden bei Patient:innen mit neu diagnostizierter PMR häufiger zu einer glukokortikoidfreien Remission bis Woche 52 führte als Placebo plus Glukokortikoide [41]. Die auf dem EULAR-Kongress 2026 vorgestellten Daten der randomisierten Phase-III-Studie REDUCE-PMR‑1 mit 116 Patient:innen erreichten für den primären Endpunkt der glukokortikoidfreien Remission nach 52 Wochen keine statistische Signifikanz. Gleichwohl zeigten sich über mehrere sekundäre Endpunkte konsistente numerische Vorteile zugunsten von Rituximab, insbesondere hinsichtlich der Remissionsraten, der kumulativen Glukokortikoiddosis und der Zeit bis zur glukokortikoidfreien Remission. Eine abschließende Bewertung der klinischen Relevanz dieser Ergebnisse und ihrer potenziellen Konsequenzen für zukünftige Therapieempfehlungen sollte jedoch erst nach Vorliegen der Vollpublikation erfolgen [42].

#### Reduktion der Glukokortikoidtherapiedauer durch den Einsatz von DMARDs

Bei Kombinationstherapien aus Glukokortikoiden und DMARDs sollte eine Reduktion der Glukokortikoiddauer angestrebt werden. Unter der Therapie mit IL-6-Rezeptor-Inhibitoren oder Rituximab ist eine Verkürzung der Glukokortikoidtherapie auf etwa 4 Monate möglich, wohingegen unter Methotrexat ein Ausschleichschema über 6 bis 8 Monate versucht werden kann [2].

### Nichtmedikamentöse Maßnahmen

Ergänzend zur medikamentösen Behandlung wird insbesondere bei älteren oder gebrechlichen Patient:innen ein individuelles Bewegungs- und Trainingsprogramm gemäß der S2e-Leitlinie empfohlen. Dieses soll dazu beitragen, die körperliche Leistungsfähigkeit zu erhalten und muskuläre Einschränkungen zu verringern, allerdings gibt es dafür nur unzureichende Evidenz.

### Laufende klinische Studien zur Therapie der PMR

Januskinase(JAK)-Inhibitoren wie Tofacitinib (NCT06172361) befinden sich derzeit in klinischer Prüfung. Trotz positiver Ergebnisse aus kleineren randomisierten Studien (z. B. Baricitinib [43]) sind sie aufgrund der bislang limitierten Evidenzlage noch nicht in die aktuelle Leitlinie implementiert worden.

Auch konventionelle synthetische DMARDs werden derzeit in prospektiven Studien evaluiert, darunter PMRLEFRCT (NCT03576794) zur Untersuchung von Leflunomid sowie die STERLING-PMR-Studie (ISRCTN17828080) mit Methotrexat und Leflunomid.

### PMR-Fast-Track-Programme

Fast-Track-Kliniken für die RZA wurden bereits in einigen Kliniken im deutschsprachigen Raum implementiert [44]. Zwei retrospektive Studien haben die Rolle eines Fast-Track-Managements bei Patient:innen mit PMR untersucht. Nach telefonischer Kontaktaufnahme von Allgemeinmediziner:innen mit dem rheumatologischen Zentrum erfolgte die Vorstellung der Patient:innen in der Fast-Track-Klinik innerhalb von 3 Tagen. Die Implementierung dieser Fast-Track-Programme zur koordinierten Betreuung durch Primär- und Sekundärversorgung führte dabei nachweislich zu einer Reduktion der Hospitalisierungsraten, einer verkürzten Zeit bis zur Diagnosestellung sowie zu niedrigeren Glukokortikoidstartdosen [45, 46].

## Schlussfolgerung

Die Polymyalgia rheumatica ist eine der häufigsten entzündlich-rheumatischen Erkrankungen im höheren Lebensalter, stellt jedoch weiterhin eine diagnostische und therapeutische Herausforderung dar. Differenzialdiagnostische Überlegungen, eine relevante Überlappung mit der RZA sowie die Notwendigkeit einer langfristigen Glukokortikoidtherapie mit potenziell erheblichen Nebenwirkungen unterstreichen die klinische Komplexität der Erkrankung.

Glukokortikoide stellen zwar noch immer die zentrale Erstlinientherapie der PMR dar, da sie die entzündungsbedingten Schmerzen und die Morgensteifigkeit meist rasch lindern, sollten jedoch wegen möglicher Nebenwirkungen so niedrig dosiert und so kurz wie möglich eingesetzt werden.

Die IL-6-Rezeptor-Inhibition stellt derzeit die Therapie der ersten Wahl zur Glukokortikoideinsparung bei der PMR dar. Methotrexat und Rituximab können gemäß S2e-Leitlinie als alternative Therapieoptionen erwogen werden, sind jedoch für diese Indikation nicht zugelassen. Positive Phase-III-Studiendaten zu Secukinumab erweitern das therapeutische Spektrum und lassen eine baldige Zulassung erwarten. Ebenfalls vielversprechende Ergebnisse in klinischen Studien zeigten JAK-Inhibitoren, ihre zukünftige Bedeutung bleibt jedoch aufgrund der bislang fehlenden Zulassung und Leitlinienempfehlung noch offen. Daher verschiebt sich das therapeutische Paradigma zunehmend von einer Monotherapie mit Glukokortikoiden hin zu einer differenzierten, risikoadaptierten Behandlungsstrategie mit konventionellen und biotechnischen Immunsuppressiva.

Zentral für eine optimierte Versorgung ist eine frühzeitige rheumatologische Abklärung sowie eine **präzise Patient:innenstratifizierung**

präzise Patient:innenstratifizierung

unter Berücksichtigung des PMR-RZA-Spektrums und anhand individueller Risikoprofile für Rezidive und therapieassoziierte Komplikationen. Die Integration von Bildgebung und serologischen Markern wird dabei voraussichtlich eine entscheidende Rolle spielen. Insgesamt entwickelt sich die PMR damit zunehmend von einer vermeintlich „einfach zu behandelnden“ Erkrankung zu einem komplexen Krankheitsbild, dessen Management primär in die rheumatologische Fachversorgung gehört.

## Fazit für die Praxis

- Eine frühzeitige rheumatologische Abklärung der PMR ist für eine korrekte Diagnose essenziell.
- Die Diagnose beruht auf der typischen Klinik, erhöhten Entzündungsparametern und dem Ausschluss relevanter Differenzialdiagnosen.
- Glukokortikoide bleiben die zentrale Erstlinientherapie, sollten jedoch so rasch wie möglich und so langsam wie nötig ausgeschlichen werden. Engmaschige Kontrollen des Therapieerfolgs sind notwendig, um Rezidive frühzeitig zu erkennen und Nebenwirkungen der Therapie zu minimieren.
- Bei erhöhtem Risiko für glukokortikoidassoziierte Komplikationen oder Rezidive sind glukokortikoidsparende Strategien, insbesondere IL-6-Rezeptor-Blocker, frühzeitig zu erwägen.
- Prognostische Faktoren und Aktivitätsscores unterstützen die individuelle Risikostratifizierung und Therapieanpassung im Verlauf.

## Data Availability

Alle dieser Arbeit zugrunde liegenden Daten sind in diesem Artikel enthalten.

## CME-Fragebogen

Bei wie viel Prozent der Patient:innen mit aktiver Polymyalgia rheumatica sind die Entzündungszeichen (CRP, BSG) normwertig?

1–3 %

20 %

40 %

60 %

80 %

Welche Form der Anämie ist bei Polymyalgia rheumatica häufig?

Hämolytisch

Megaloblastisch

Normozytär

Makrozytär

Aplastisch

Welches der folgenden bildgebenden Verfahren hat die höchste Sensitivität bei der Diagnostik der Polymyalgia rheumatica?

Computertomographie

Duplexsonographie

Native Röntgenbilder

Kapillarmikroskopie

Magnetresonanztomographie

Was ist die übliche Prednisolon-Tagesdosis, die bei Erstdiagnose einer Polymyalgia rheumatica eingesetzt werden sollte?

10 mg

20 mg

40 mg

60 mg

90 mg

Ein 72-jähriger Patient erhält eine Therapie mit Glukokortikoiden wegen Polymyalgia rheumatica. Nach initialer Besserung treten erneut typische Beschwerden auf. Welche therapeutische Maßnahme ist zunächst am ehesten indiziert?

Dauerhaftes Absetzen der Therapie ohne weitere Anpassung

Erhöhung der Glukokortikoiddosis auf die zuletzt wirksame Dosis

Wechsel auf nichtsteroidale Antiphlogistika als Monotherapie

Sofortige operative Therapie der Schultergelenke

Beendigung der Therapie bei Rezidivsymptomatik

Welches Medikament ist für die glukokortikoidsparende Behandlung der Polymyalgia rheumatica bei Patient:innen, die auf Kortikosteroide unzureichend angesprochen haben oder bei denen es während der Kortikosteroidreduktion zu einem Rezidiv kommt, aktuell zugelassen?

Infliximab

Sarilumab

Upadacitinib

Cyclophosphamid

Azathioprin

Die Mobilität in welchem Gelenk geht in den Polymyalgia rheumatica-Aktivitätsscore ein?

Knie

Hüfte

Schulter

Handgelenk

Metakarpophalangealgelenk II

Wie häufig treten Rezidive unter Reduktion von Prednisolon auf?

< 10 %

10–20 %

40–60 %

80 %

> 90 %

Eine 74-jährige Patientin mit gesicherter Polymyalgia rheumatica entwickelt neu aufgetretene Kopfschmerzen, Kauschmerzen und transiente Visusminderung. Die Entzündungsparameter sind weiterhin erhöht. Welche Konstellation ist am ehesten zutreffend?

Entwicklung einer degenerativen Kiefergelenkarthrose im Rahmen der Grunderkrankung

Wahrscheinliche Transformation in eine rheumatoide Arthritis

Überlappung mit einer Riesenzellarteriitis im Rahmen eines Polymyalgia rheumatica-RZA-Spektrums

Typische Nebenwirkung einer Glukokortikoidtherapie

Primäre ophthalmologische Erkrankung ohne systemische Relevanz

Das ^18^F‑FDG-PET-CT wird in seltenen Fällen als Bildgebung bei der Diagnose der Polymyalgia rheumatica eingesetzt. Was ist der typische Befund?

Periartikuläre Anreicherung an Schultern und Hüften

Periartikuläre Anreicherung an Knien und oberem Sprunggelenk (OSG)

Artikuläre Anreicherung an Schultern und Hüften

Artikuläre Anreicherung an Knien und OSG

Anreicherung der juxtaartikulären Gefäße
